# Blunt Trauma Resulting in Testicular Evisceration: A Case Report

**DOI:** 10.7759/cureus.14019

**Published:** 2021-03-21

**Authors:** Roderick Olivas, Syed Uddin, Bhani Chawla Kondal, Avinash Chenam

**Affiliations:** 1 General Surgery, Riverside Community Hospital - HCA Healthcare, Riverside, USA; 2 Surgery, Riverside Community Hospital - HCA Healthcare, Riverside, USA; 3 Trauma, Riverside Community Hospital - HCA Healthcare, Riverside, USA; 4 Urology, Riverside Community Hospital - HCA Healthcare, Riverside, USA

**Keywords:** scrotum, testicle, blunt trauma, testicular injury, scrotal injury, scrotal hematoma, testicular hematoma, testicular evisceration, scrotal rupture

## Abstract

Scrotal and testicular injuries are uncommon injuries, accounting for only a fraction of all trauma. Blunt scrotal trauma is accompanied by testicular rupture in up to 50% of cases. We present a rare case of scrotal rupture with evisceration of a viable, intact testicle after a motor vehicle accident. The patient’s presentation, associated injuries, operation, and post-operative course are described. In brief, this is a case of a 69-year-old male who sustained multiple rib, pelvic, and right femur fractures in addition to scrotal injury after a motor vehicle accident. He was taken quickly to the operating room for the scrotal rupture, and his testicle was successfully replaced and scrotal laceration repaired. He did well post-operatively. This case represents one of the few accounts of this particular injury in the literature.

## Introduction

Scrotal and testicular trauma are uncommon injuries, accounting for less than 1% of overall trauma in the United States [1-3]. Scarcity of these injuries is attributed to the location and mobility of the scrotum [3]. A recent study using the National Trauma Data Bank found over an eight-year period that testicular and scrotal trauma had a prevalence of only 0.23% [1]. Similar rates have been reported in military literature [4]. A significant portion of cases is due to blunt traumatic mechanisms; up to 85% is cited in the literature [1,5]. Common causes include traffic accidents, most frequently motorcycle-related, industry accidents involving heavy machinery, and contact sports [1,3,6]. Testicular rupture occurs in nearly 50% of all blunt scrotal trauma, and delayed treatment can damage testicular functionality or even result in testicular loss [7]. We present a unique injury of scrotal rupture with complete evisceration of a viable testicle without testicular rupture due to blunt trauma. Such cases have rarely been reported previously [8]. This study was approved by the hospital Institutional Review Board, and need for informed consent was waived.

## Case presentation

**Figure 1:**
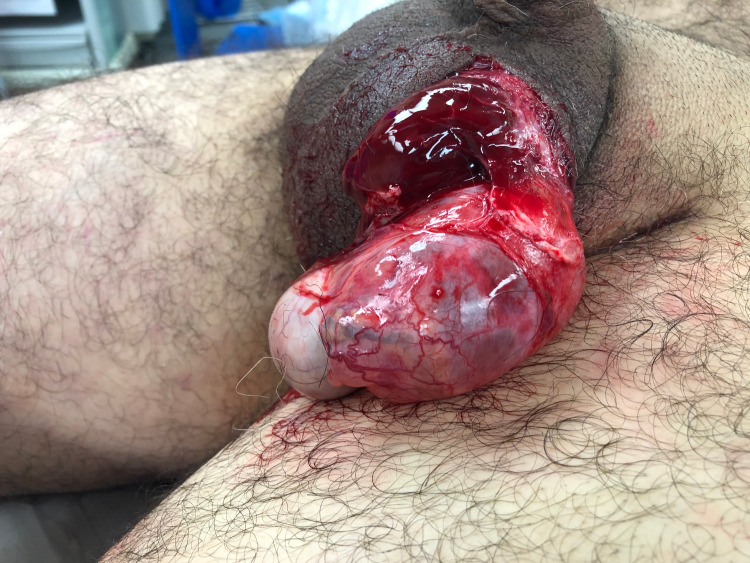
Scrotal rupture with evisceration of the testicle and hematoma

A 69-year-old man with an unknown past medical history presented to the emergency department after being involved in a motor vehicle collision. He rear-ended a trailer travelling at freeway speed and self-extricated. The patient was hemodynamically stable, but further history was limited on arrival due to uncontrollable pain causing agitation and he was quickly intubated. Left scrotal laceration with complete testicular evisceration was noted on initial survey (Figure 1). After further evaluation and imaging, he was also found to have the following associated injuries: right fourth-eighth rib fractures with an associated hemopneumothorax, left third-ninth rib fractures, bilateral superior and inferior pubic rami fractures, a right acetabulum fracture, and a segmental right femoral shaft fracture with fracture points in proximal and distal thirds. A right-sided chest tube was placed in the emergency department and then the patient was taken directly to the operating room for scrotal exploration by urology followed by femur fixation by orthopedic surgery. In the operating room, the testicle clinically appeared viable with an anterior hematoma. Characterization utilizing AAST (American Association for the Surgery of Trauma) Organ Injury Scaling was scrotal injury grade V and testicular injury grade II [9]. The hematoma was evacuated, the testicle was replaced in the scrotal sac and pexied, and the scrotal laceration was repaired. Post-operatively, the patient recovered well with an uncomplicated course. He was extubated on hospital day 3 and discharged in stable condition on hospital day 10. The patient has since followed up in the office and continues to do well.

## Discussion

Scrotal rupture with evisceration of a viable testicle is an unusual injury caused by blunt trauma. Mechanism of injury in these cases is believed to be a shearing force pressing the scrotum against a bony surface (such as the femur or pelvis), sacrificing the scrotum’s mobility and low position and causing a majority of the force to be transferred directly to the scrotum and testis [10]. AAST Injury Scales have been created to characterize both scrotal trauma and testicular trauma [9]. Other than degloving injuries, which are also quite rare, isolated high-grade scrotal injuries with low-grade testicular injuries have not often been described in the literature [8]. Thus, this particular case is unique. Evaluation of scrotal trauma typically relies on scrotal ultrasound as first line and being a fast and noninvasive way to assess for testicular compromise [10]. When ultrasound cannot provide a definitive answer or, as in this case, testicular viability can be assessed visually, the next diagnostic step is surgical exploration of the scrotum. Other indications for surgery include clinical findings of testicular injury, disruption of the tunica albuginea on ultrasound, and/or absence of blood flow on sonograms with Doppler studies [2]. The American Urological Association (AUA) recommends early scrotal exploration in all patients suspected of testicular rupture to avoid complications such as testicular loss, infection, chronic pain, infertility, and altered self-image [11]. Early surgical intervention has been linked with a decreased morbidity and improved likelihood of maintaining testicular viability [12].

## Conclusions

Scrotal and testicular injuries are unusual, accounting for only a fraction of trauma. We present an interesting and unique case of scrotal rupture and high-grade injury, with complete evisceration of a minorly injured, viable testicle due to blunt trauma with multiple associated injuries. This patient was managed by prompt surgical scrotal exploration and recovered uneventfully with preservation of the involved testicle. Similar cases have rarely been described in the literature.
